# Role of the Gene *ndufs*8 Located in Respiratory Complex I from *Monascus purpureus* in the Cell Growth and Secondary Metabolites Biosynthesis

**DOI:** 10.3390/jof8070655

**Published:** 2022-06-22

**Authors:** Xinru Cai, Song Zhang, Jia Lin, Yaxu Wang, Fanyu Ye, Bo Zhou, Qinlu Lin, Jun Liu

**Affiliations:** National Engineering Research Center of Rice and Byproduct Deep Processing, College of Food Science and Engineering, Central South University of Forestry and Technology, Changsha 410004, China; caixinru1668@163.com (X.C.); zhangsong941110@126.com (S.Z.); linjijia3570@163.com (J.L.); wyx526539@163.com (Y.W.); a15927345477@126.com (F.Y.); zhbo78@163.com (B.Z.); t20081475@csuft.edu.cn (Q.L.)

**Keywords:** *M. purpureus*, *ndufs*8, electron transport chain, secondary metabolism, growth

## Abstract

Our previous work revealed that the anabolism of *Monascus* secondary metabolites is closely related to cofactor metabolism. In this study, we have further investigated the regulation mechanisms of respiratory complex I in response to the cell growth and secondary metabolite biosynthesis of *M. purpureus*. The results showed that downregulating the mRNA level of gene *ndufs*8 in *M. purpureus* sharply increased the secondary metabolites biosynthesis, cell growth and glucose consumption rates at the fermentation metaphase; slightly increased the colony diameter and biomass, and dramatically changed the mycelia morphology; and decreased the tolerances to environmental factors (especially H_2_O_2_). It also significantly inhibited the enzymes activities of respiratory complex I, III and superoxide dismutase, but stimulated that of complex II, IV and peroxidase, leading to an increase in reactive oxygen species (ROS) level and a decrease in ATP concentration. Furthermore, transcriptome analysis revealed that the mRNA levels of genes involved in respiratory chain, tricarboxylic acid cycle, and fatty acid degradation were downregulated, but those in the citrinin and *monascus* pigment biosynthesis and related pathways were upregulated. These data revealed that complex I plays a vital role in regulating the cell growth and secondary metabolism of *Monascus* via changing the intracellular ROS and ATP levels.

## 1. Introduction

The *Monascus* secondary metabolites, *monascus* pigments (MPs) and monacolin K (MK) are famous for their particular biological functions, such as coloring, antibacterial, antioxidation, anti-inflammatory, regulating cholesterol and anti-cancer [1,2], and are being widely utilized by food industries worldwide, especially China, Japan, and other Southeast Asian countries [3]. However, the secondary metabolite, citrinin (CT) is nephrotoxic and hepatotoxic to animals, which limits its application of MPs and MK productions, especially in Europe and America [4,5]. Among these secondary metabolites, the composition of MPs is complex and numerous. According to the tone, MPs are mainly classified into orange MPs (O-MPs, absorption at 460–480 nm), red MPs (R-MPs, absorption at 490–530 nm) and yellow MPs (Y-MPs, absorption at 330–450 nm) [6], including six kinds of classic hydrophobic MPs and four kinds of classically red-toned hydrophilic MPs [7].

*Monascus* species are oxygenic strains; theoretically, respiratory activity exerts an enormous function on cell growth and metabolism. The biosynthesis pathways of MPs and CT are similar, synthesized using acetyl-CoA and malonyl-CoA as the substrates through PKS (type I) pathway and FAS pathway. Especially, O-MPs are reduced to Y-MPs contents through a hydrogenation reaction dependent on NADH or NADPH, produced to R-MPs contents through an amination reaction involved in amino acid participation [8] or a facile non-enzymatic spontaneous reaction [9]. It has been reported that cofactor metabolism plays a vital role in manipulating the targeted product biosynthesis, such as butanol [10,11], spinosad and pseudoaglycone [12], antrodin C [13] and MPs [14]. Additionally, cofactor NADH participates in 43 and 65 reactions in *Escherichia coli* and *Saccharomyces cerevisiae*, respectively [15]. Complex I (NADH-quinone oxidoreductase, EC:7.1.1.2) plays a central role in energy production by respiratory chain and provides 40% of H^+^ force for ATP biosynthesis [16]. Hence, NADH could affect the metabolic flux redistribution and regulate the cell growth and biosynthesis of products. It follows then that disruption of the mitochondrial electron transfer chain (ETC) or respiratory chain would regulate the cell growth and the biosynthesis of MPs and CT.

Our previous study investigated the effects of cofactor metabolism on MPs and CT productions in *M. purpureus* through the application of different cofactor engineering strategies, including disruption of the expression of gene *monascus*_4971 [17]. The data showed that the production of Y-MPs, O-MPs, R-MPs and CT increased by 54.86%, 36.68%, 9.10%, and 5.88% in submerged batch-fermentation (SBF) by a heterokaryotic recombination strain *M. purpureus* Δ4971, respectively. Besides, disruption of MPs and CT biosynthesis by knockout genes *pig*R and *pks*CT changed the morphology, as well as the level of intracellular cofactor NADH and NADPH [18]. However, the analysis of functional annotation of gene *monascus*_4971, the effect of disturbed ETC on glucose consumption, MPs and CT biosynthesis and cell growth, the exact molecule mechanism of regulation effect have not been introduced in detail.

## 2. Materials and Methods

### 2.1. Microorganisms and Culture Conditions

The wild-type strain *M. purpureus* LQ-6 (named WT strain, CCTCC M 2018600, China Central for Type Culture Collection (CCTCC), Wuhan, China) [19] and the mutant strain *M. purpureus* Δ4971 generated by disrupting the mRNA level of gene *monacsus*_4971 from *M. purpureus* LQ-6 in our laboratory [17] were stored on potato dextrose agar (PDA) medium with 50 μg/mL of geneticin (G418 sulfate) as needed at 4 °C were used for experiments.

Stored strains were inoculated on fresh PDA medium at 30 °C for seven days in a constant temperature incubator for activation, and then the hyphae were picked from the PDA medium containing the activated *M. purpureus* strains by using an inoculating loop to place on PDA medium at 30 °C. When the agar plates were covered with fully-grown culture of *M. purpureus*, spore suspension (adjusted to 10^6^ spores/mL) was prepared by the addition of sterilized water to the PDA medium and sweeping on them.

### 2.2. Sensitivity Test

Ten μL of the generated spore suspension of *M. purpureus* LQ-6 and *M. purpureus* Δ4971 were inoculated into the middle of various PDA plates (diameter = 90 mm) with different concentrations of sodium chloride (0 M, 0.5 M, 1.0 M, 1.5 M, 2.0 M), hydrogen peroxide (0 mM, 2 mM, 4 mM, 6 mM, 8 mM), and ethanol (0%, 3% (*v*/*v*), 6% (*v*/*v*), 8% (*v*/*v*), 10% (*v*/*v*)), and cultured at 30 °C for eight days in a constant temperature incubator to daily measure the changes in colony diameter.

### 2.3. Image Analysis

The colony morphologies of *M. purpureus* LQ-6 and Δ4971 on PDA plates and mycelium from SBF were monitored using a Motic compound microscope (BA200, Nuremberg, Germany) and a biological microscope (Olympus, Tokyo, Japan). Besides, the mycelium was collected and fixed with 2.5% glutaraldehyde at 4 °C for 12 h, samples were observed using scanning electron microscopy (SEM) (SU8010, Hitachi, Japan) by Beijing Zhongke Baice Technology Co., Ltd. (Beijing, China).

### 2.4. Submerged Batch-Fermentation and Kinetics

Ten mL of spore suspension were inoculated into 100 mL of the fermentation medium (glucose 80 g/L, yeast extract 2.5 g/L, malt extract 2.5 g/L, peptone 2.5 g/L, K_2_HPO_4_ 5 g/L, CaCl_2_ 0.1 g/L, MgSO_4_·7H_2_O, 0.5 g/L, FeSO_4_·7H_2_O 0.01 g/L, ZnSO_4_·7H_2_O 0.01 g/L and MnSO_4_·7H_2_O 0.03 g/L, pH = 5) in 250 mL conical flasks at 30 °C and agitated at 180 rpm in the dark for 10 days for the SBF [19], and the fermentation kinetics were carried out in 1000 mL conical flasks with 400 mL of fermentation medium; the culture conditions were the same as the SBF. The fermentation curves were fitted by using the Boltzmann equation.

(1) $$Y=A_{2}+\frac{A_{1}-A_{2}}{1+\exp\left(\frac{x-x_{0}}{\mathrm{dx}}\right)}$$

where Y represents the response values of the evaluation object, A_1_ represents the initial value of the evaluation object, A_2_ represents the final value of the evaluation object, x represents the fermentation time, x_0_ and dx represent the coefficients of the equation.

### 2.5. Enzyme Activity Detection

SBF broth at 84 h and 168 h were centrifuged at 8085× *g* and at 4 °C for 10 min to collect *Monascus* mycelium, then 0.1 g of the collected mycelium were broken in nine-fold volume of ice-cold phosphate buffered solution (PBS) (50 mM, pH = 6.8) to generate the mycelium lysate. Then, the lysate was centrifuged at 8085× *g* and at 4 °C for 10 min to obtain the supernatant. The activities of peroxidase (POD, EC:1.11.1.7), total kinds of superoxide dismutase (T-SOD, EC1.15.1.1), ETC complex I, II and III IV were determined by using the assay kits (Nanjing Jiancheng Bioengineering Institute, Nanjing, China) [20]. Protein concentrations were measured using the conventional Bradford assay. Reactive oxygen species (ROS) was measured according to the protocol by using the assay kit (E004-1-1, Nanjing Jiancheng Bioengineering Institute, Nanjing, China). The concentrations of ATP were detected by using the assay kit (A095-1-1, Nanjing Jiancheng Bioengineering Institute, Nanjing, China).

### 2.6. Determination of Metabolites and Calculations

The detection and calculation methods of glucose concentration, MPs, citrinin and biomass were performed as our previous studies [7,17,19]. We uniformly aspirated 10 mL from the fermentation broth to measure the OD value of the supernatant at wavelengths of 505 nm, 470 nm, and 420 nm, and calculated the extracellular pigments (red, orange, and yellow) color values. After centrifugation, the lower mycelium was extracted with 70% (*v*/*v*) ethanol at 60 °C for 2 h, and 70% ethanol was used as a blank control. At the same time, the OD value was measured and converted to obtain the intracellular color value. The total biomass was expressed as the dry cell weight (DCW), and the residual glucose concentration was measured according to the 3,5-dinitrosalicyclic acid method, high-performance liquid chromatography was employed to analyze the citrinin production, as previously described.

### 2.7. Transcriptomic Analysis

The methods of total RNA extraction, RNA sequencing (RNA-seq) library construction, and high-throughput sequencing were described in our previous study [7]. Six samples (three duplicate samples of *M. purpureus* LQ-6 and *M. purpureus* Δ4971) were collected at 84th h during SBF, and high-throughput sequencing was performed on an Illumina Hiseq 4000 platform by Gene Denovo Biotechnology Co., Ltd. (Guangzhou, China) (https://www.genedenovo.com (accessed on 23 June 2021)).

Differentially expressed genes (DEGs) in mutant strain *M. purpureus* Δ4971 relative to parent strain *M. purpureus* LQ-6 were subjected to Gene Ontology (GO) functional analysis and Kyoto Encyclopedia of Genes and Genomes (KEGG) pathway enrichment analysis. The genes in transcripts with false discovery rate (FDR) < 0.05 and |log2FC| > 1 were set as the DEGs.

### 2.8. Validation of Gene Expression Levels via RT-qPCR

Glyceraldehyde-3-phosphate dehydrogenase (GAPDH) was selected as the reference gene for RT-qPCR, and the primers used in this study were listed in Table S1. RNA samples were treated with RNase-free DNaseI (Thermo Fisher Scientific, Waltham, MA, USA) following the manufacturer’s protocol to remove the residual genomic DNA. Besides, the first-strand cDNA was synthesized using oligo-dT primers and *EasyScript*^®^ Reverse Transcriptase (TransGen Biotech, Beijing, China). RT-qPCR was performed using the *TransStart*^®^Green qPCR SuperMix UDG (TransGen Biotech, Beijing, China) according to the manufacturer’s instructions.

### 2.9. Data Analysis

Each experiment was repeated at least three times; the numerical data are presented as the mean ± SD in the figures, and statistical analyses were performed using the SPSS Statistics 23 (SPSS, Chicago, IL, USA). Data were analyzed by one-way ANOVA, and tests of significant differences were determined by using the Ducan multiple comparison or Student’s *t*-test at *p* < 0.05.

### 2.10. Accession Numbers

The raw RNA-seq data of the present study were deposited into the NCBI database with an accession number of PRJNA740360. The gene *monacsus*_4971 was located in the genome sequences of *M. purpureus* LQ-6, which have been deposited into the NCBI database with an accession number of PRJNA503091.

## 3. Results and Discussion

### 3.1. Functional Annotation of Gene monascus_4971

**Figure 1:**
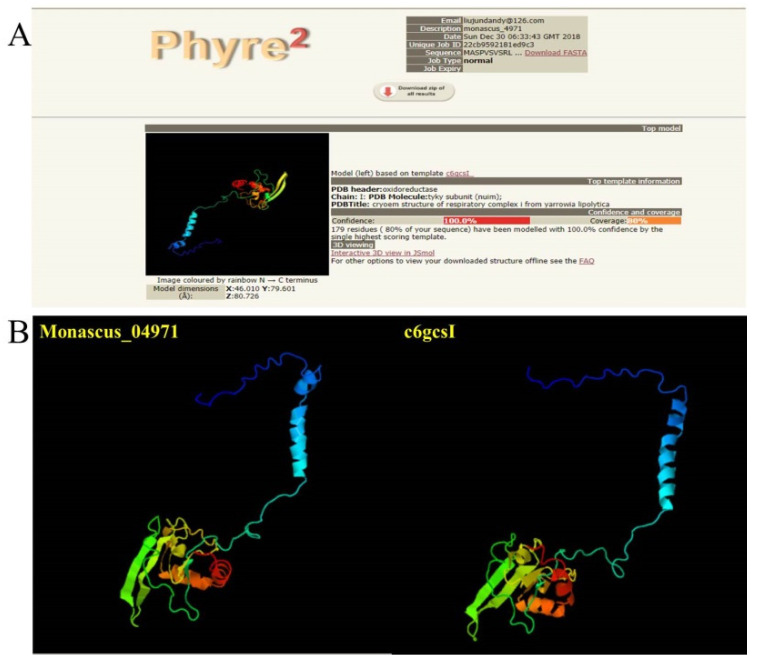
The predicted result of the tertiary structure of protein encoding by gene *monascus*_4971 using the phyre2 web server. The overview (**A**) and the tertiary structure of protein (**B**).

From the genomic analysis of *M. purpureus* LQ-6, the gene *monascus*_4971 with the total number of length of 1180 bp, encoding a protein with the total number of length of 225 aa, which was identified as 23 kDa subunit of NADH-quinone oxidoreductase (22% Query Cover, 88.21% Identity) in *Aspergillus terreus* NIH2624 (accession: XM_001212139.1) by searching the NCBI-blastn, and NADH dehydrogenase Fe-S protein subunit 8 (*ndufs*8, 23 kDa subunit of the mitochondrial complex I) (100% Query Cover, 100% Identity) in *M. purpureus* HQ1 (accession: TQB76855.1) by searching the NCBI-blastp. The 23 kDa subunit of the human mitochondrial respiratory complex I is 72% identical to the *Rhodobacter capsulatus nuo*I counterpart [21]. Furthermore, the phyre2 web server predicted the spatial structure of the protein encoding by gene *monascus*_4971, and the result showed that it was almost same as the spatial structure of protein 6gcsI with 100% confidence and 80% coverage (Figure 1). In fact, the protein data bank showed that 6gcs (NADH: ubiquinone oxidoreductase) is the respiratory complex I from *Yarrowia lipolytica* (*Candida lipolytica*), and 6gcsI (229 aa, 25.68 kDa) is the NADH: ubiquinone oxidoreductase chain I (https://www.ebi.ac.uk/pdbe/entry/pdb/6gcs/protein/9 (accessed on 10 October 2018)), and the domain homologous to the gene *nuim* (UniProtKB-Q9UUT8, 33-229 aa, coverage: 83%) encoding NADH: ubiquinone oxidoreductase 23 kDa subunit (https://www.uniprot.org/uniprot/Q9UUT8 (accessed on 1 May 2020)). In addition, InterPro annotation indicated that NADH-quinone oxidoreductase, chain I (IPR010226) represents the subunit I (*nuo*I, one of 14 subunits, A to N) of the NADH-quinone oxidoreductase complex I in bacteria (https://www.ebi.ac.uk/interpro/entry/InterPro/IPR010226/ (accessed on 3 October 2020)).

**Figure 2:**
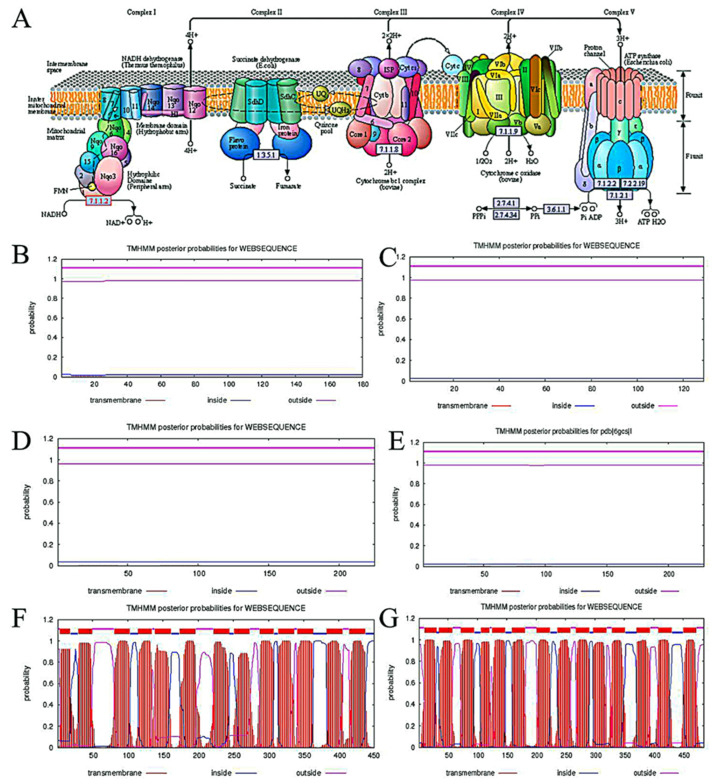
The ETC structure (**A**) and the predicted transmembrane helices of gene *nuo*I by TMHMM Server v. 2.0 in *E. coli* K-12 (**B**), *C. beijerinckii* NCIMB 8052 (**C**), *Y. lipolytica* (**D**)*,* gene *monacsus*_4971 in *M. purpureus* LQ-6 (**E**), that of gene *nuo*M (**F**) and *nuo*N (**G**) in *E. coli* K-12, respectively.

It is well known that complex I (NADH-quinone oxidoreductase, EC:7.1.1.2), one of the largest membrane proteins, presents an L-shaped structure with a hydrophobic arm (embedded in the membrane) and a hydrophilic peripheral arm (protruded into the mitochondrial matrix) [16,22,23]. In addition, the mitochondrial and bacterial enzymes contain equivalent redox components and structure [22], protein NDUFS8 (called Nqo9) is located in the hydrophilic peripheral arm (outside of membrane) [22]. Hence, CBS Prediction Servers-Protein function and structure (TMHMM Server v. 2.0, http://www.cbs.dtu.dk/services/TMHMM-2.0 (accessed on 22 March 2021)) was applied for further determining the function of gene *monascus*_4971 and the number of subunit in complex I. Figure 2A shows the structure and location of ETC, subunits Nqo1 (ndufv1), Nqo2 (ndufv2), Nqo3 (*ndufs*1), Nqo4 (*ndufs*2), Nqo5 (*ndufs*3), Nqo6 (*ndufs*7), Nqo9 (*nuo*I/*ndufs*8), Nqo15 and Nqo16 of mitochondrial complex I are located in the hydrophilic peripheral arm, Nqo12 (*nuo*L), Nqo13 (*nuo*M), Nqo14 (*nuo*N), Nqo11 (*nuo*K), Nqo10 (*nuo*J), Nqo7 (*nuo*A) and Nqo8 (*nuo*H/ND1) are located in the hydrophobic arm. Based on the above results, the transmembrane structure of gene *nuo*I in *E. coli* K-12 (as the representative of prokaryotic and aerobic microorganism) and *Clostridium beijerinckii* NCIMB 8052 (as the representative of prokaryotic and anaerobic microorganism), gene *6gcsI* in *Yarrowia lipolytica* (as the representative of eukaryotic and aerobic microorganism) were analyzed by TMHMM Server v. 2.0. As shown in Figure 2F,G, gene *nuo*M and *nuo*N (located in hydrophobic arm) in *E. coli* K-12 has 13 and 14 predicted transmembrane helices, respectively. However, gene *nuo*I in different strains has no predicted transmembrane helices, as well as gene *monascus*_4971 (Figure 2B–E). In addition, the expected number of amino acids in transmembrane helices is 0.28102, 0.00145, 0.04973, 0.03387 and 0.03387 in *E. coli* K-12, *C. beijerinckii* NCIMB 8052, *Y. lipolytica, M. purpureus* HQ1 and *M. purpureus* LQ-6, respectively, which is significantly lower than 18 (Table 1). Therefore, we determined that gene *monascus*_4971 is *ndufs*8 (traditionally known as *nuo*I in bacteria) located in mitochondrial complex I.

### 3.2. Determination of the Transformant Strain

**Figure 3:**
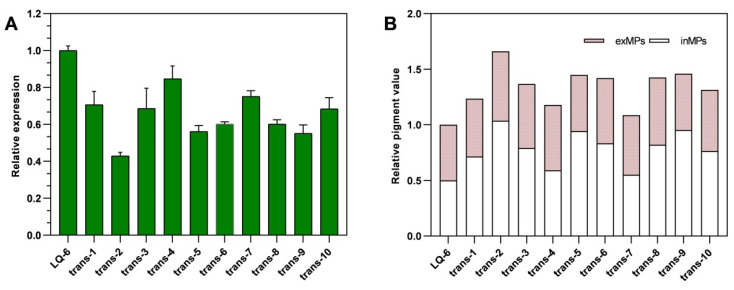
The mRNA relative level of gene *monascus*_4971 in mutant strains (**A**), the relative value of total monascus pigments production by different mutant strains (**B**).

Regrettably, we did not find the homozygotes by PCR validation, all the transformant strains were heterokaryotic. We speculated that the enzyme NADH: ubiquinone oxidoreductase 23 kDa subunit encoded by gene *ndufs*8 in the mitochondrial complex I plays a crucial role in the growth of *M. purpureus*. Thus, ten random transformant strains (trans-1 to trans-10) were selected to evaluate the mRNA level of gene *monascus*_4971 by RT-qPCR at 7th day and the MPs production through SBF for 10 days. Figure 3 shows that the mRNA level of gene *monascus*_4971 located in the ten mutant strains were both downregulated compared with that in the parent strain LQ-6. Specially, we found that the mRNA level of gene *monascus*_4971 was contrary to the MPs biosynthesis (as the representative of secondary metabolites), the strain trans-2 revealed the lowest mRNA level of gene *monascus*_4971 (downregulated by 60% compared with that in LQ-6, Figure 3A), but produced the highest MPs value (enhanced by 70% compared with that by LQ-6, Figure 3B). However, the mutant trans-2 had poorer stability of MPs production through multiple passages. Then, one random transformant (named *M. purpureus* Δ4971) was chosen in our laboratory.

### 3.3. Effect of ndufs8 Gene Located in Mitochondrial Complex I on Monascus Growth and Morphology

**Figure 4:**
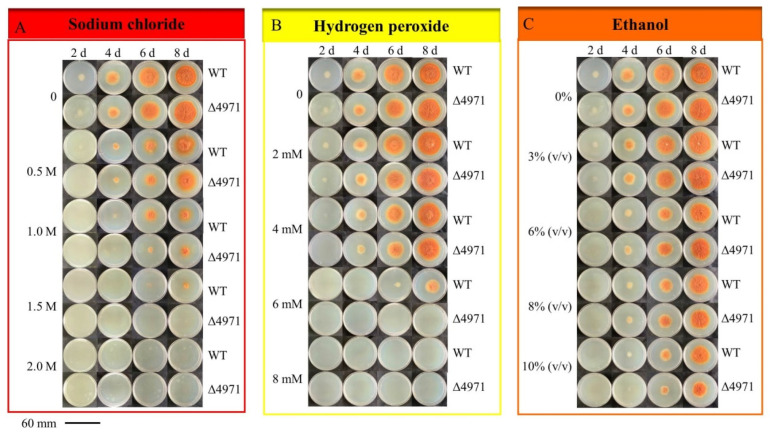
The sensitivities of parent strain *M. purpureus* LQ-6 (WT) and the mutant strain *M. purpureus* Δ4971 cultured on PDA medium to NaCl (**A**), H_2_O_2_ (**B**), ethanol (**C**).

It is well known that the intracellular energy metabolism exerts an enormous function on cell normal growth to various organisms. In mitochondria, the proton electrochemical potential (ΔE_H+_) generated from proton translocation with NADH as the H^+^ donor, depending on complex I, III and IV, is used for ATP synthesis. Thus, we investigated the effect of *ndufs*8 gene located in mitochondrial complex I on the cell growth and morphology of *M. purpureus*. From Figure 4, the mycelium morphology on PDA solid medium is almost identical between WT and the mutant strains. Besides, we found that the sensitivity of the mutant strain to NaCl was significantly increased compared with that of the parent strain, and no visible growth of *M. purpureus* Δ4971 was apparent in response to 1.5 M of NaCl (Figure 4A); when the concentration of H_2_O_2_ was higher (more than 4 mM), it suddenly became sensitive to H_2_O_2_, and no visible growth of *M. purpureus* Δ4971 was apparent in response to 6 mM of H_2_O_2_, but the colony diameters of the LQ-6 strain in response to 6 mM of H_2_O_2_ were 6.75 mm and 25.15 mm at 6th and 8th day, respectively (Figure 4B); the WT strain has a strong tolerance to ethanol, but the germination time was prolonged from the 4th day to the 6th day in the presence of 10% (*v*/*v*) ethanol after disrupting the expression level of *ndufs*8 gene (Figure 4C).

**Figure 5:**
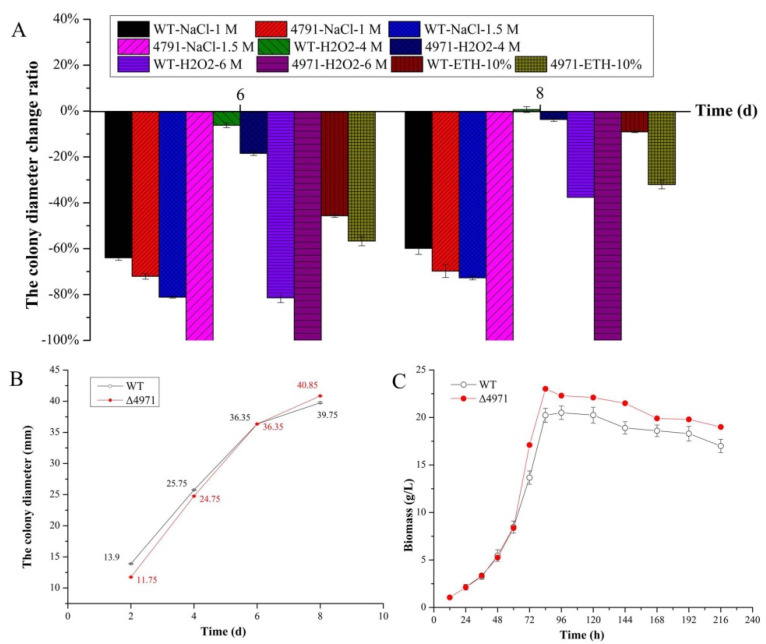
The change ratio of colony diameters of WT and Δ4971 strains on PDA medium with compounds supplementation (**A**), the colony diameters of them cultivated on PDA medium (**B**), the biomass of them during submerged batch-fermentation (**C**).

**Figure 6:**
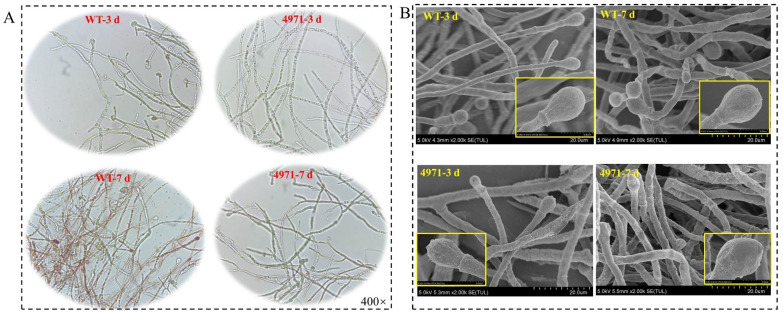
Image analysis of the mycelial morphologies of *M. purpureus* LQ-6 (WT) and *M. purpureus* Δ4971 during submerged batch-fermentation by using biological microscope (**A**) and scanning electron microscopy (**B**).

Furthermore, the colony diameter of WT and Δ4971 strain was suddenly decreased by 81.43% and 100% on PDA medium with the addition of 6 mM of H_2_O_2_ at 6^th^ day, respectively, and it was decreased by 37.61% and 100% at 8^th^ day during the process of cultivation. Obviously, the colony diameters of WT and Δ4971 strain were decreased by 45.67% and 56.67% at 6th day, 9.06% and 32.07% at 8th day under the stress of 10% (*v*/*v*) of ethanol, respectively (Figure 5A). These data illustrate that H_2_O_2_ exhibits the strongest inhibited ability to disturb cell growth among these three environmental cofactors, and it could be further deteriorated after disrupting the expression of *ndufs*8 gene in *Monascus* spp. However, the curve of colony diameter shows that disruption of the *ndufs*8 gene expression exhibited insignificant changes in the germination rate of spores and growth rate of *Monascus* on solid medium (Figure 5B). Besides, we also found that the rate of cell growth suddenly enhanced at 60th h during SBF by *M. purpureus* Δ4971, and the biomass of *M. purpureus* Δ4971 and LQ-6 was 16.72 g/L (enhanced by 30.52%) and 12.81 g/L at 72th h during SBF, respectively. However, there was insignificant difference in the biomass of *M. purpureus* Δ4971 and LQ-6 at the end of fermentation (Figure 5C). Besides, the number of conidias and branches structures in the hypha of *M. purpureus* Δ4971 was decreased compared with that of the parent strain, but reversed to the length at the 3rd day during the cultivation (Figure 6A). SEM exhibited that the changes of mycelium morphology of them were insignificant at the 3rd day during SBF, but the parent strain had noticeable depressions (numerous rough and folded hypha) at the 7th day compared with that of the mutant strain, which also had numerous spiculate protrusions (Figure 6B). These results state that disruption of the electron transfer through mitochondrial complex I has a vital influence on the mycelium morphology and differentiation of *Monascus*.

**Figure 7:**
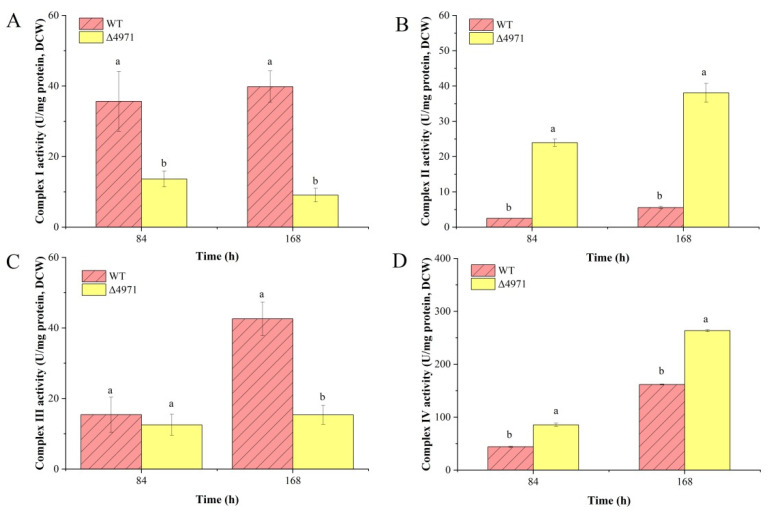
Comparison of the activities of enzyme complex I (**A**), complex II (**B**), complex III (**C**) and complex IV (**D**) of the parent strain and mutant strain during submerged batch-fermentation. Different letters (a and b) in the same column indicate a significant difference (*p* < 0.05).

**Figure 8:**
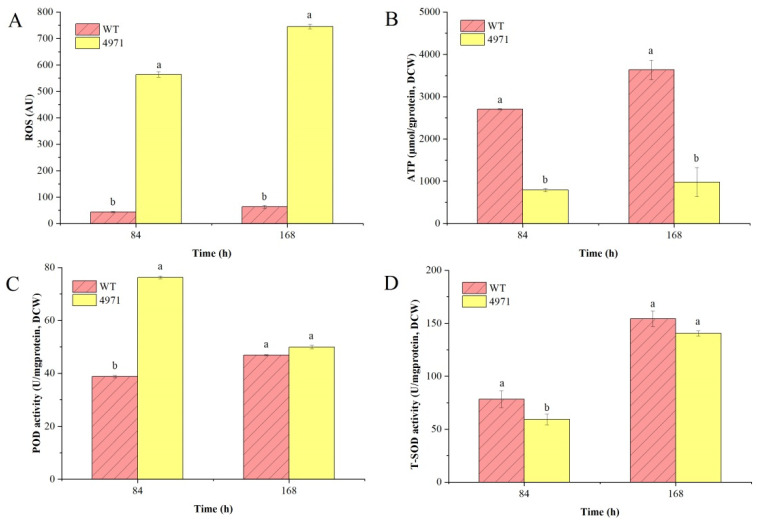
Comparison of intracellular ROS level (**A**), ATP concentration (**B**), and the enzyme activities of peroxidase (**C**) and superoxide dismutase (**D**) of the parent strain and mutant strain during submerged batch-fermentation. Different letters (a and b) in the same column indicate a significant difference (*p* < 0.05).

The formation of ROS, including H_2_O_2_, hydroxyl radical (HO•) and superoxide anions (O^2−^) in cells, would be a harmful common physiological stress in the habitat of filamentous fungi [13]. Besides, it has been reported that ROS has a vital effect on the biomass, mycelium morphology and secondary metabolism biosynthesis [24]. Chen et al. reported that exogenous addition of rotenone, a kind of complex I inhibitor, could significantly increase the ROS level in cell [25]. In this study, we disturbed the electron transfer by suppressing the expression level of *ndufs*8 gene located in mitochondrial complex I. The results showed that the activity of complex I was significantly inhibited during SBF process (Figure 7A), and that of complex III (Figure 7C) was decreased by 18.61% at the initial stage of fermentation (84 h) and 63.92% at the fermentation metaphase (168 h), but that of complex II (Figure 7B) and complex IV (Figure 7D) were dramatically stimulated after disrupting the mRNA level of *ndufs*8 gene. It has been reported that ROS production by ETC was closely associated with complex I and complex III in *S. cerevisiae* [26]. Generally, there are two ETC pathways, NADH-complex I-CoQ-complex III-complex IV-O_2_ (NADH-dependent ETC), and FADH_2_-complex II-CoQ-complex III-complex IV-O_2_ (FADH_2_-dependent ETC), but the former dominates the electron flow. Chemiosmotic hypothesis is well known, ETC is coupled with ATP synthesis by a proton pump or ATPase with the oxidation of NADH to NAD^+^ for ATP production. During this process, 2.5 M of ATP could be produced when NADH donates a pair of electrons. Thus, we further measured the levels of ROS and ATP and predictably, the ROS level in the mutant strain was increased by more than 5-fold and the ATP concentration was decreased by more than 3-fold compared with that in the parent strain (Figure 8A,B). Besides, there are many efficient enzymes, including SOD, POD, catalase (CAT) and glutathione reductase (GR), which are closely collaborated to eliminate or reduce the intracellular ROS level and form the antioxidant defense machinery [27]. In this study, the higher intracellular ROS level of mutant strain may dramatically stimulate the upregulation of the POD activity at the initial stage of fermentation (Figure 8C). However, it was not expected that the POD activity in the mutant strain markedly decreased to 49.92 U/mg protein at the middle stage of fermentation (168 h), and it was insignificant to that (46.89 U/mg protein) in the parent strain. We speculated that the reduced activity of enzyme POD activity or the ability of ROS elimination may be regulated by one or more factors at the middle stage of fermentation for intracellular ROS accumulation, and stimulating the secondary metabolism. In addition, the activity of T-SOD was almost insignificant between these two strains (Figure 8D), which resulted in a further enhancement of the ROS level at the middle stage of fermentation, and illustrated that T-SOD did not play a positive role in reducing the intracellular ROS level. Similarly, Zhou et al. also found that SOD-Mn has an inconsequential function in eliminating ROS in *Monascus* [20]. Besides, the comparatively higher ROS level may stimulate the cell growth of *Monascus*. These data explained the reasons for these changes in cell differentiation and cell sensitivity to environmental factors after disrupting the electron transfer through mitochondrial complex I.

### 3.4. Effect of ndufs8 Gene on the Monascus Secondary Metabolism

**Figure 9:**
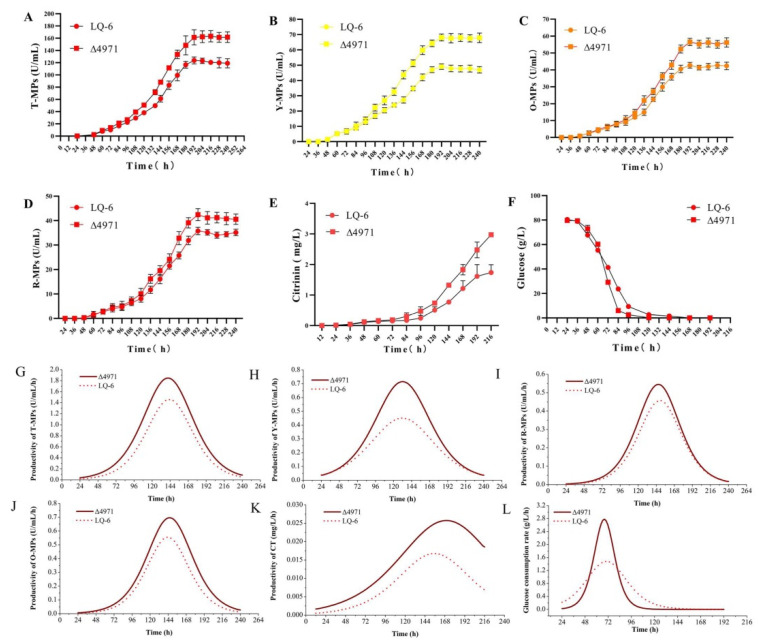
Submerged batch-fermentation kinetics analysis of *M. purpureus* LQ-6 and *M. purpureus* Δ4971. The biosynthesis of T-MPs (**A**), Y-MPs (**B**), O-MPs (**C**), R-MPs (**D**) and citrinin (**E**); glucose consumption (**F**); the productivity of T-MPs (**G**), Y-MPs (**H**), O-MPs (**I**), R-MPs (**J**) and citrinin (**K**); the rate of glucose consumption (**L**).

The compared fermentation kinetics of *M. purpureus* Δ4971 and LQ-6 shows that the biosynthesis of secondary metabolites, including MPs and CT, and glucose consumption sharply increased after downregulating the expression of gene *ndufs*8 in SBF (Figure 9). Figure 9A–D shows that the production of T-MPs (total production of MPs), Y-MPs, O-MPs and R-MPs by mutant strain reached the maximum at 192 h during the SBF process, with an increase of 30.47%, 38.74%, 32.22% and 18.61% compared with those from the parent strain, respectively. Besides, the fermentation kinetic curves were simulated by using the Boltzmann model. The results showed that the correlation coefficient (R^2^) between the test value and the predicted value of the model were approximately 0.98 (Table 2), which could well reflect the kinetic characteristics of the SBF process by *M. purpureus* Δ4971 and LQ-6. From Table 2, we could obtain that the maximum production of T-MPs, Y-MPs, O-MPs, R-MPs and CT by *M. purpureus* Δ4971 would reach 169.02 U/mL, 70.88 U/mL, 42.75 U/mL, 58.18 U/mL and 3.89 mg/L, with an increase of 34.01%, 39.23%, 18.01%, 32.97% and 94.59% compared with those from the parent strain, respectively. These data illustrated that disrupting the complex I or downregulating the expression of *ndufs*8 gene could stimulate the *Monascus* secondary biosynthesis, but has a stronger effect on the biosynthesis of Y-MPs and CT. Moreover, we also found that the glucose consumption rate suddenly increased from 60th h during SBF after downregulation of the mRNA level of gene *ndufs*8 in *M. purpureus* LQ-6 (Figure 9F). Furthermore, the results of productivity and consumption rate curves (Figure 9G–L) showed that the productivities of Y-MPs and CT, and the rate of glucose consumption by the mutant and parent strains were significantly different. The maximum of Y-MPs productivity reached 0.72 U/mL/h from 0.45 U/mL/h (increased by approximately 60%) at 133.09 h after downregulating the mRNA level of gene *ndufs*8, and that of CT reached 0.026 mg/L/h at 168.61 h from 0.017 mg/L/h at 152.12 h during the SBF process. Obviously, the glucose consumption rate of *M. purpureus* Δ4971 was much higher than that of the parent strain at the range of 60~80 h during SBF, and the maximum rate of glucose consumption reached 2.78 g/L/h, with an increase of 87.84%, (Figure 9L) which also explained the phenomenon of the changes in the cell growth rate of *M. purpureus* Δ4971 and LQ-6 during SBF.

In our previous study, the intracellular NADH and NADPH levels were higher than those after downregulation of the mRNA level of *ndufs*8 gene in the parent strain, and explained the relevance of the biosynthesis of MPs and CT and the cofactor metabolism [17]. In this study, we also speculated that the increased intracellular ROS level could stimulate the biosynthesis of *Monascus* secondary metabolites, especially Y-MPs and CT. Hu et al. utilized H_2_O_2_ supplementation or addition of complex I inhibitor rotenone during SBF, causing ROS accumulation and triggering the biosynthesis of the targeted product antrodin C [13]. Besides, it has been reported that H_2_O_2_ supplementation could induce oxidative conditions or oxidation reduction potential (ORP) changes, causing Y-MPs overproduction via upregulation of the mRNA levels of relative genes [14]. These reports indicated that although intracellular ROS accumulation can damage cell growth, it can also simulate the biosynthesis of target product by regulating the expression of relative genes. The leakage of electrons from mitochondrial ETC has been described as the major source of ROS production. Thus, many researches focus on the disruption of respiratory complex I to regulate the biosynthesis of target product by changing the environmental ORP and ROS levels [13,14,17]. In the present study, suppression of *ndufs*8 gene expression led to the ROS accumulation, causing the upregulated biosynthesis of MPs and CT.

### 3.5. Regulation Mechanisms of ndufs8 Gene in Monascus

**Figure 10:**
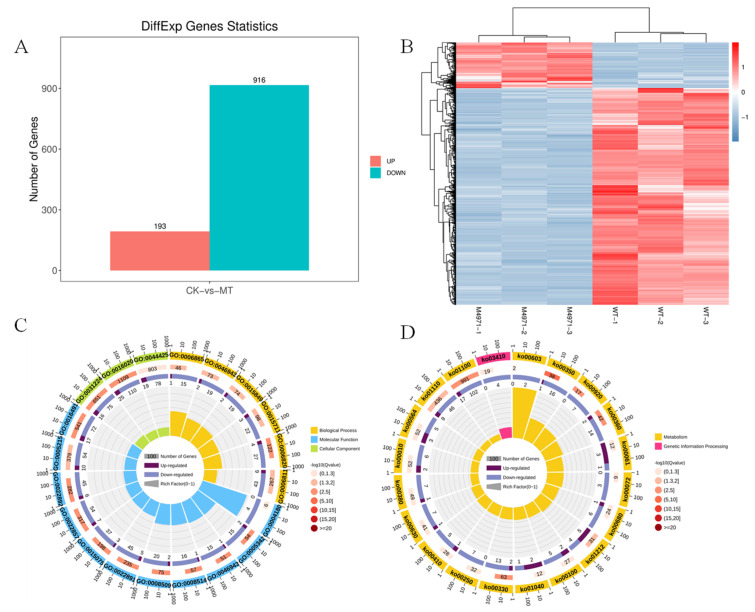
Comparison of gene expression data between the mutant strain *M. purpureus* Δ4971 (MT) and the wild-type strain *M. purpureus* LQ-6 (WT). Number of DEGs in MT compared to WT (**A**), the DEGs clustering (**B**), GO enrichment analysis (**C**) and KEGG enrichment analysis (**D**).

To reveal the exact molecule mechanism of regulating effect on secondary metabolism and cell growth, a comparative transcriptome analysis was carried out. As Figure 10 shows, there were 1109 DEGs (193 genes were upregulated and 916 genes were downregulated, Figure 10A,B) identified by *M. purpureus* Δ4971 (M4971) compared with the parent strain (WT). The top 20 terms of GO enrichment analyses show that the DEGs were mainly involved in oxidoreductase activity (GO:0016491) and transmembrane transporter activity (GO:0022857) in the ontology of molecular function, membrane (GO:0016020) and intrinsic component of membrane (GO:0031224) in the ontology of cellular component, amino acid transport (GO:0006865) and anion transport (GO:0006820) in the ontology of biological process (Figure 10C). Besides, the top 20 pathways of KEGG pathway enrichment analyses show that the DEGs were mainly involved in metabolic pathways (ko01100), biosynthesis of secondary metabolites (ko01110), fatty acid metabolism (ko01212), and amino acid metabolism (ko00350 and ko00330) (Figure 10D). These data revealed that carbon metabolism, energy metabolism and secondary metabolism were regulated after disrupting the expression of *ndufs*8 gene in *M. purpuresus*. Hence, the pathways of glycolysis, tricarboxylic acid cycle (TCA), ETC, polyketide synthesis (PKS), fatty acid synthesis (FAS), fatty acid degradation (FAD), MPs and CT biosynthesis were in-deep analyzed in this study.

**Figure 11:**
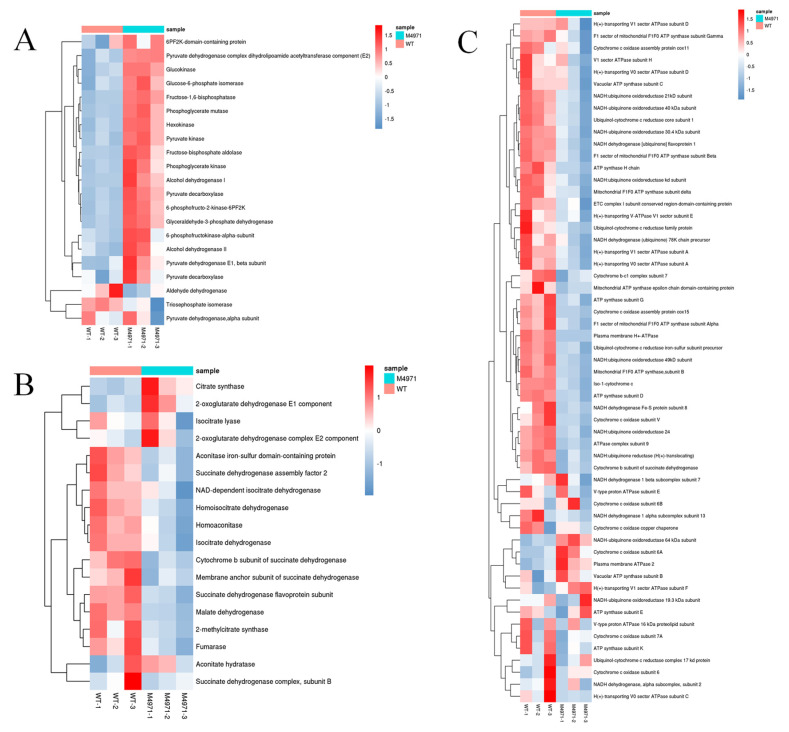
Comparison of gene expression in glycolytic pathway (**A**), tricarboxylic acid cycle (**B**), respiratory chain (**C**) between the mutant strain *M. purpureus* Δ4971 (MT) and the wild-type strain *M. purpureus* LQ-6 (WT).

As shown in Figure 11A, DEGs involved in glycolysis (also called EMP) were almost upregulated, including the key enzymes glucokinase, 6-phosphofructo-2-kinase (6PF2K) and pyruvate kinase. In addition, the enzyme pyruvate dehydrogenase complex was significantly upregulated, same as the alcohol dehydrogenase I and alcohol dehydrogenase II, but the aldehyde dehydrogenase and triosephosphate isomerase was significantly (log_2_^FC^ = −1.95) and weakly (log_2_^FC^ = −0.33) downregulated, respectively. Inhibition of aldehyde biosynthesis may stimulate ethanol biosynthesis, leading to another kind of abiotic stress, but would also be beneficial for the cell growth and secondary metabolism of *Monascus*. This deduction has been verified and analyzed in details in our previous study [28], and in which, we also summarize some research achievements on the addition of ethanol in the SBF process to enhance the production of secondary metabolites by *Monascus* strains. During the EMP pathway, we speculated that the downregulated triosephosphate isomerase may promote the conversion of dihydroxyacetone phosphate to lipid compounds (FAS upregulated); this would consume NAD(P)H and be beneficial to the balance of intracellular energy. Besides, the mRNA level of glucose-6-phosphate 1-dehydrogenase (G6PDH), the key enzyme in the pentose phosphate pathway (PPP), was significantly upregulated by 32.16%. However, the TCA pathway was almost weakly inhibited after the mRNA level of gene *ndufs*8 was decreased in the parent strain, such as the isocitrate dehydrogenase, homoisocitrate dehydrogenase, succinate dehydrogenase complex and malate dehydrogenase (Figure 11B). Furthermore, compared with *M. purpureus* LQ-6, the NADH: ubiquinone oxidoreductase complex I, cytochrome b, cytochrome c oxidase (complex III) and H (+)-transporting ATPase complex in respiratory chain were both completely downregulated in the mutant strain (Figure 11C). It has been reported that the mitochondrial transmembrane potential loss and the changes in distribution are closely related to the ROS production, and complex I and III are the well-known sites of the production of superoxide radicals, which can be further reduced by SOD dismutation to H_2_O_2_ [27]. Many researchers have reported that H_2_O_2_ can act as a key regulator in a broad range of physiological processes, including cell growth and development [29,30], and in secondary metabolism [13]. The transcriptome data showed that the mRNA levels of genes encoding SOD, POD, CAT, alternative oxidase (AOX), glutathione-S-transferase (GST) and GR were insignificant expressed (slightly downregulated) in the mutant strain, but the POD activity was significantly upregulated and it reversed to that of T-SOD. It is well-known that the mRNAs regulated at the transcription level were not always regulated in the same direction at the translation level. Poor correlation might be attributed to the differential regulation at mRNA and protein levels, such as a generally longer half-life of protein than mRNA and different regulations at the post-transcriptional, translational, and post-translational levels for proteins [31] However, a certain amount of oxidative or abiotic stress remained in this situation, which is beneficial to the stimulation of the cell growth and secondary metabolism.

Gene *ndufs*8 encoding NADH: ubiquinone oxidoreductase 23 kDa subunit is located in oxidative phosphorylation of energy metabolism (NADH and ATP). In this study, the ATP generation through oxidative phosphorylation pathway (respiratory chain/ETC) was disturbed, causing to the glycolysis (substrate level phosphorylation) upregulated for supplying the required energy to cell growth and metabolism, these explained the reason of increased glucose consumption rate in the mutant strain. In addition, combined with the results of enzyme activity assay, it stated that *ndufs*8 plays an impotent role in electron transfer (especially through complex I and III) and ATP generation. However, higher mRNA levels of pyruvate dehydrogenase promoted to the acetyl-CoA generation, but inhibited the TCA pathway which led to the excess production of acetyl-CoA, which were advantage to FAS, MPs and CT biosynthesis.

**Figure 12:**
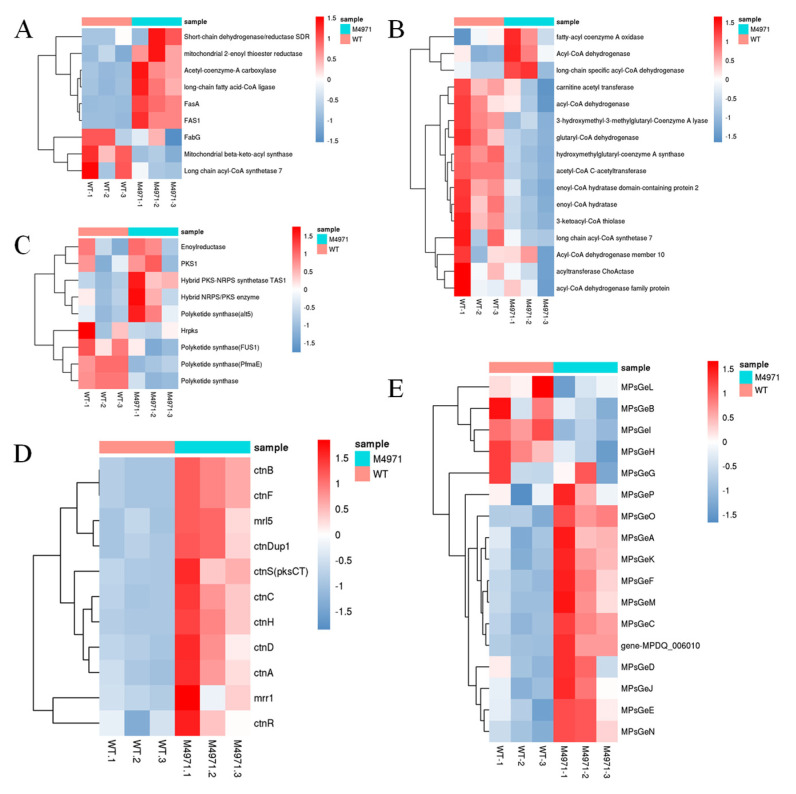
Comparison of gene expression in fatty acid synthesis pathway (**A**), fatty acid degradation pathway (**B**), polyketone synthesis (**C**), citrinin biosynthesis gene cluster (**D**), monascus pigments biosynthesis gene cluster (**E**) between the mutant strain *M. purpureus* Δ4971 (MT) and the wild-type strain *M. purpureus* LQ-6 (WT).

We further investigated the mRNA level of MPs and CT biosynthetic gene clusters, and the related pathways including FAS, FAD and PKS. The comparative transcriptome showed that FAS and PKS were mostly upregulated while it reversed to the FAD pathway (Figure 12A–C), which illustrated that the excess production of acetyl-CoA from the upregulated glycolysis preferred to flow into the FAS pathway and the inhibited FAD pathway, conversed into the substrate ketone for MPs and CT biosynthesis. In addition, the expression of MPs and CT biosynthetic gene clusters were almost completely upregulated, especially the CT biosynthetic gene clusters (ctnB-R, approximately 1.5-fold; Figure 12D). Except four genes (MPsGeL (ankyrin repeat protein), MPsGeB (transcription factor), MPsGeI (transcription factor), MPsGeH (enoyl reductase), the others in MPs biosynthetic gene clusters were approximately 1.5-fold higher compared with that of parent strain (Figure 12E). To validate the data of transcriptome, the expression level of DEGs related to MPs and CT biosynthesis and ETC (including gene *ndufs*8) were analyzed by RT-qPCR in both *M. purpureus* Δ4971 and LQ-6 strains. Fortunately, the results of RT-qPCR were almost consistent with that of the transcriptome analysis (Table 3), which illustrated that the gene *ndufs*8 located in ETC complex I indeed plays an important role in regulating the secondary metabolism of *M. purpureus*.

## 4. Conclusions

**Figure 13:**
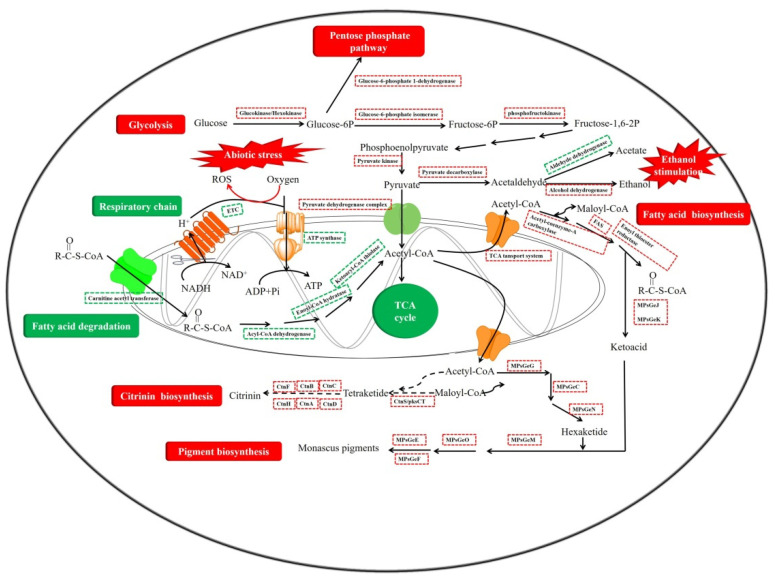
Overview of disrupted metabolism pathways from transcriptomic in the mutant strain *M. purpureus* Δ4971 compared with that in *M. purpureus* LQ-6. Green represents the downregulated genes and pathways, and red represents the upregulated genes and pathways.

Overall, in this study, we found that disruption of the ETC through altering the mRNA level of *ndufs*8 gene located in complex I in *Monascus* could result in ROS accumulation and ethanol production, causing the pathways of TCA cycle and FAD to be inhibited, but the upregulated pathways of substrate level phosphorylation (including EMP and PPP), FAS, PKS, CT and MPs biosynthesis (Figure 13). It is the first report demonstrating that NADH: ubiquinone oxidoreductase 23 kDa subunit (*ndufs*8) or ETC complex I plays an important role in global regulating the cell growth and secondary metabolism of *M. purpureus* in detail, and reveals the exact molecule mechanism by using the method of comparative transcriptome analysis.

## Figures and Tables

**Table 1:** Comparison of the TMHMM results of gene *nuo*I in different strains.

Parameters	*Escherichia coli* K-12	*Clostridium beijerinckii* NCIMB 8052	*Yarrowia lipolytica*	*M. purpureus* HQ1	*M. purpureus* LQ-6 (in this Study)
Length of the protein sequence	180	129	229	225	225
Number of predicted TMHs	0	0	0	0	0
Exp number of AAs in TMHs	0.28102	0.00145	0.04973	0.03387	0.03387
Exp number, first 60 AAs	0.27782	0.00013	0.01368	0.0259	0.0259
Total prob of N-in	0.02778	0.02446	0.03104	0.03812	0.03812
Outside	1~180	1~129	1~229	1~225	1~225

Number of predicted TMHs: The number of predicted transmembrane helices. Exp number of AAs in TMHs: The expected number of amino acids in transmembrane helices. If this number is larger than 18 it is very likely to be a transmembrane protein (OR have a signal peptide). Exp number, first 60 AAs: The expected number of amino acids in transmembrane helices in the first 60 amino acids of the protein. If this number is more than a few, you should be warned that a predicted transmembrane helix in the N-term could be a signal peptide. Total prob of N-in: The total probability that the N-term is on the cytoplasmic side of the membrane. Possible N-term signal sequence: a warning that is produced when “Exp number, first 60 AAs” is larger than 10.

**Table 2:** Boltzmann Fitting equations and the correlation coefficient R^2^ of fermentation kinetics of *M. purpureus* Δ4971 and LQ-6.

Strain	Object	A_1_	A_2_	X_0_	dx	R^2^
*M. purpureus* LQ-6	T-MPs	5.78753	126.1309	142.87989	20.60122	0.98793
	Y-MPs	0.05763	50.90419	131.63568	28.16209	0.98421
	R-MPs	0.99312	36.22905	148.89972	19.07795	0.99059
	O-MPs	1.95999	43.753	143.40017	18.79606	0.98846
	CT	0.01955	1.99955	155.31582	29.51801	0.99583
	Glucose consumption	83.92365	0.12268	70.16283	14.13061	0.99751
*M. purpureus* Δ4971	T-MPs	4.58268	169.02451	141.06142	22.22952	0.99514
	Y-MPs	0.20583	70.8763	131.97551	24.66908	0.99326
	R-MPs	1.28916	42.75523	146.74975	18.9883	0.99005
	O-MPs	1.75071	58.17946	145.81863	20.22318	0.99312
	CT	−0.0636	3.89083	169.87953	38.3883	0.99891
	Glucose consumption	79.3281	0.07146	67.93989	7.09183	0.99898

**Table 3:** The validation of transcriptome data by RT-qPCR.

GeneID	Description	CT		Log_2_ Ratio (Δ4971/LQ-6)
		LQ-6	Δ4971	
Gen-MPDQ_006632	*ndufs*8	20.24	22.23	−0.410
Gen-MPDQ_006025	MpigA	24.58	24.23	0.695
Gen-MPDQ_006009	MpigP	23.60	24.23	0.125
Gen-MPDQ_006023	MpigC	20.30	19.58	0.587
Gen-MPDQ_006022	MpigD	20.94	20.98	0.192
Gen-MPDQ_006016	MpigJ	19.84	19.22	0.514
Gen-MPDQ_006018	MpigH	24.97	26.61	−0.486
Gen-MPDQ_006019	MpigG	22.56	25.31	−0.005
Gen-MPDQ_003567	CtnsS(pksCT)	25.64	24.07	1.40
Gen-MPDQ_003569	ctnsB	21.53	19.42	1.67
Gen-MPDQ_003571	ctnsD	23.22	21.10	1.38
Gen-MPDQ_003574	ctnsC	23.35	21.17	1.47
Gen-MPDQ_001039	Hybrid PKS-NRPS synthet-aseTAS1	21.31	19.05	0.889
Gen-MPDQ_008094	Hybrid NRPS/PKS enzyme	24.12	22.18	0.151
Gen-MPDQ_001369	NADH deydrogenase	19.61	22.98	−0.797
Gen-MPDQ_003356	ETC complex Ⅰ subunit conserved region-domain-containing protein	20.59	23.07	−0.531
Gen-MPDQ_002668	NADH:ubiquinone oxidoreductase	19.89	22.83	−0.844
Gen-MPDQ_004264	NADH dehydrogenase	22.45	24.63	−0.490
Gen-MPDQ_000146	NADH-ubiquinone oxidoreductase	18.61	20.74	−0.589

## Data Availability

The data presented in this study are available upon request from the corresponding author. The raw RNA-seq data of the present study was deposited into the NCBI database with an accession number of PRJNA740360. The gene monascus_4971 located in the genome sequences of *M. purpureus* LQ-6, which have been deposited into the NCBI database with an accession number of PRJNA503091.

## Supplementary Materials

The following supporting information can be downloaded at: https://www.mdpi.com/article/10.3390/jof8070655/s1, Table S1: Primers used in this study. Table S2: The expression level of genes involved in MPs biosynthesis. Table S3: The expression level of genes involved in citrinin biosynthesis. Table S4: The expression level of genes involved in fatty acid synthesis. Table S5: The expression level of genes involved in fatty acid degradation. Table S6: The expression level of genes involved in PKS pathway. Table S7: The expression level of genes involved in respiratory chain. Table S8: The expression level of genes involved in glycolysis pathway. Table S9: The expression level of genes involved in TCA pathway. Table S10: The expression level of genes encoding enzymes SOD, POD, CAT, AOX, GST and GR.
