# Supplementary material for: Role of the Gene ndufs8 Located in Respiratory Complex I from Monascus purpureus in the Cell Growth and Secondary Metabolites Biosynthesis
Source: J Fungi (Basel). 2022 Jun 22;8(7):655. doi: 10.3390/jof8070655 (PMC9319538; doi:10.3390/jof8070655)
Supplement: Supplementary file 1 [file jof-08-00655-s001.zip › Table S1.pdf]

Table S1. Primers used in this study.

Primers	Sequences（5’→3’）	Reference
RT-GAPDH- F	CGATGCCCAGTAATTATTAGGAC	This study
RT-GAPDH-R	CTGTAGCGCCTGACCTCGTA	This study
RT-4971-F	CGCTATCCTACTGGTGAAGAAC	This study
RT-4971-R	CTTCGGCCTCGATGGTAATAG	This study
RT-6632-F	CGCTATCCTACTGGTGAAGAAC	This study
RT-6632-R	CTTCGGCCTCGATGGTAATAG	This study
RT-6025 -F	GCAGCATCTACCCAGCTATATT	This study
RT-6025 -R	CCACAGTCAATCCAGCTCTT	This study
RT-6009 -F	AAGGGCCTCTTGACCTTTG	This study
RT-6009 -R	CCTGAGACGAGTTCTTCGTATTT	This study
RT-6023 -F	CGCAGCTAGGCATTGTCATA	This study
RT-6023 -R	GAAGAACAAGAGCAGACCTACC	This study
RT-6022 -F	GAGGCCCTGATGGTTTAAGT	This study
RT-6022-R	CGTTTCTCGGAGGTGAGATAG	This study
RT-6016 -F	CGACCCGTCTGTCAAGTTTAT	This study
RT-6016-R	TCATCTTGCACAGGTCATCC	This study
RT-6018 -F	CTGGAGGAGCATCGGAAAC	This study
RT-6018-R	CCACAACATCCTTCGTCTTGA	This study
RT-6019 -F	CCAGGGCCGCAAGTTTAT	This study
RT-6019-R	GATCCACGAGACGAAGAAGATG	This study
RT-3567-F	CGGTATGTTCCAGGTTGAGATAG	This study
RT-3567 -R	GGTCATGTAGCTCTGGGATAAAG	This study
RT-3569 -F	CCACTTCGACTGCTCCATTAT	This study
RT-3569 -R	ATGGCTCCAAAGTCCGTATG	This study
RT-3571 -F	CAGAAGCACAGCCATGAATTG	This study
RT-3571 -R	CACCAGAAACCCAACATCAAAG	This study
RT-3574 -F	GGTCAGTCTGATGGGCTTTAC	This study
RT-3574 -R	ACACTAGGAGCGTCGAAGATA	This study
RT-1039-F	GGTCCTTGCAATGGGTTAGA	This study
RT-1039 -R	AGCCATATACCCTGCTGATTG	This study
RT-8094 -F	CTGGCTCGTGGATACCTTAAC	This study
RT-8094 -R	AGAGCATTGGGCGTCTTATC	This study
RT-1369 -F	TTGACATGCTCCAGGAACTC	This study
RT-1369 -R	CTCAGGTCGGAAGTGTCTAATC	This study
RT-3356 -F	GAAGAGGCCCAATTCAACAAAG	This study
RT-3356 -R	GGCTCATTCTCCCACTTCAT	This study
RT-2668 - F	CTATGGCGATTGGTGGGATAA	This study
RT-2668 -R	GTGTACTGCTCGACGCTAAA	This study
RT-4294-F	GGAGGCGGATGCTATTCTTT	This study
RT-4294 -R	CTCACCTACAAGGGCAATCTC	This study
RT-0146 -F	CGAACTCACCGTCTATGTTTCT	This study
RT-0146-R	AGTAATGTCGGAGACCTGAGTA	This study
