# Supplementary material for: Role of the Gene ndufs8 Located in Respiratory Complex I from Monascus purpureus in the Cell Growth and Secondary Metabolites Biosynthesis
Source: J Fungi (Basel). 2022 Jun 22;8(7):655. doi: 10.3390/jof8070655 (PMC9319538; doi:10.3390/jof8070655)
Supplement: Supplementary file 1 [file jof-08-00655-s001.zip › Table S10.pdf]

Table S10. The expression level of genes encoding enzymes SOD, POD, CAT, AOX, GST and GR.

	Symbol	WT-1_count	WT-2_count	WT-3_count	M4971-1_count	M4971-2_count	M4971-3_count	log2(fc)
**SOD**	manganese superoxide dismutase (gene-MPDQ_003552)	1550	1670	1454	2055	2138	1653	0.212679
	Superoxide dismutase [Mn] (gene-MPDQ_002144)	1592	1836	1785	1965	2216	1815	0.098096
	superoxide dismutase copper/zinc binding protein (gene-MPDQ_007641)	625	565	478	696	643	513	0.036173
	Superoxide dismutase [Cu-Zn] (gene-MPDQ_001794)	71	86	65	107	112	81	0.322247
	Iron/manganese superoxide dismutase (gene-MPDQ_003548)	352	563	417	438	512	374	-0.11326
	Superoxide dismutase [Mn] (gene-MPDQ_002128)	5004	4893	3946	4117	4529	3625	-0.28206
	Superoxide dismutase (gene-MPDQ_001764)	51	27	49	33	36	29	-0.48598
	Manganese/iron superoxide dismutase (gene-MPDQ_000527)	819	810	791	785	677	559	-0.37404
**POD**	Peroxiredoxin Asp f3 (gene-MPDQ_004604)	3957	4042	3856	4001	4161	3301	-0.15791
	Peroxiredoxin Asp f3 (gene-MPDQ_000969)	118	127	107	134	143	105	0.00638
	mitochondrial peroxiredoxin PRX1 (gene-MPDQ_001181)	1262	1198	1102	1157	1193	965	-0.21348
	glutathione peroxidase gpx1 (gene-MPDQ_000490)	650	612	547	490	517	422	-0.44895
**CAT**	catalase-domain-containing protein (gene-MPDQ_005985)	389	434	342	168	188	199	-1.16209
	peroxisomal catalase A (gene-MPDQ_008162)	1382	1425	1253	1570	1492	1247	-0.02407
	Catalase (gene-MPDQ_002235)	364	335	329	171	258	212	-0.77953
	catalase A (gene-MPDQ_001495)	721	838	737	470	676	452	-0.62922
	Catalase B (gene-MPDQ_003968)	2438	2297	2120	2568	2026	1605	-0.26485
**AOX**	Alternative oxidase (gene-MPDQ_007869)	3966	6547	5004	12618	13502	10481	1.133732
	Alternative oxidase (gene-MPDQ_006645)	2314	2235	2623	1298	1295	1178	-1.03188
**GST**	Glutathione S-transferase (gene-MPDQ_000664)	985	1132	1178	518	522	481	-1.21701
	glutathione S-transferase (gene-MPDQ_004772)	8347	7068	5959	5270	4677	3286	-0.81437
	Glutathione S-transferase (gene-MPDQ_007961)	440	485	486	543	542	447	0.010854

	HCCA isomerase/glutathione S-transferase kappa (gene-MPDQ_003659)	62	46	53	18	32	24	-1.22137
	glutathione S-transferase (gene-MPDQ_004177)	87	75	56	92	105	80	0.234357
	glutathione S-transferase (gene-MPDQ_002347)	27	47	38	18	26	21	-0.85685
	glutathione S-transferase (gene-MPDQ_007715)	27	47	38	18	26	21	-0.85685
**GR**	Glutathione reductase (gene-MPDQ_001155)	915	1540	1151	1756	1780	1562	0.399473
