# Supplementary material for: Role of the Gene ndufs8 Located in Respiratory Complex I from Monascus purpureus in the Cell Growth and Secondary Metabolites Biosynthesis
Source: J Fungi (Basel). 2022 Jun 22;8(7):655. doi: 10.3390/jof8070655 (PMC9319538; doi:10.3390/jof8070655)
Supplement: Supplementary file 1 [file jof-08-00655-s001.zip › Table S2.pdf]

Table S2. The expression level of genes involved in MPs biosynthesis.

**Symbol**	**WT.1_count**	**WT.2_count**	**WT.3_count**	**M4971.1_count**	**M4971.2_count**	**M4971.3_count**	**log2.fc.**
MPsGeB（gene-MPDQ_006024）	45.28	34.32	41.41	35.23	33.47	29.84	-0.29634
MPsGeL（gene-MPDQ_006014）	33.08	32.08	43.05	21.8	28.88	30.53	-0.4141
MPsGeI（gene-MPDQ_006017）	336.78	321.09	350.07	225.06	246.67	226.63	-0.52937
MPsGeH（gene-MPDQ_006018）	737.38	680.25	635.6	553.11	502.56	410.06	-0.48628
MPsGeG（gene-MPDQ_006019）	81.62	75.51	75.4	77.6	81.14	72.96	-0.00516
MPsGeF（gene-MPDQ_006020）	426.55	356.73	326.93	899.76	746.5	627.55	1.034279
MPsGeE（gene-MPDQ_006021）	372.48	340.53	296.2	466.1	463.1	396.49	0.393517
MPsGeD（gene-MPDQ_006022）	781.4	696.23	681.09	884.29	852.88	728.34	0.19171
MPsGeC（gene-MPDQ_006023）	313.12	275.81	289.21	470.21	430.53	418.54	0.587228
MPsGeA（gene-MPDQ_006025）	72.36	50.69	59.97	115.14	89.51	91.59	0.694765
MPsGeP（gene-MPDQ_006009）	149.42	130.46	146.38	163.83	154.02	147.01	0.125063
gene-MPDQ_006010	185.65	177.09	175.06	304.37	264.14	264.29	0.6309
MPsGeO（gene-MPDQ_006011）	57.08	57.65	51.53	80.63	74.9	76.69	0.482051
MPsGeN（gene-MPDQ_006012）	44.86	41.3	38.43	61.1	60.44	52.61	0.483142
MPsGeM（gene-MPDQ_006013）	138.4	122.12	121.91	225.89	192.88	171.16	0.625348
MPsGeK（gene-MPDQ_006015）	66.63	50.14	56.14	108.16	90.81	86.31	0.722357
MPsGeJ（gene-MPDQ_006016）	109.76	85.11	89.9	152.05	138.6	115.98	0.513919
