# Supplementary material for: Role of the Gene ndufs8 Located in Respiratory Complex I from Monascus purpureus in the Cell Growth and Secondary Metabolites Biosynthesis
Source: J Fungi (Basel). 2022 Jun 22;8(7):655. doi: 10.3390/jof8070655 (PMC9319538; doi:10.3390/jof8070655)
Supplement: Supplementary file 1 [file jof-08-00655-s001.zip › Table S3.pdf]

Table S3. The expression level of genes involved in citrinin biosynthesis.

Symbol	WT.1_count	WT.2_count	WT.3_count	M4971.1_count	M4971.2_count	M4971.3_count	log2.fc.
ctnC（gene-MPDQ_003574）	904	700	722	2854	2229	1893	1.46544062
mrl5（gene-MPDQ_003572）	85	121	82	321	312	222	1.456969793
ctnD（gene-MPDQ_003571)	690	627	510	2204	1703	1260	1.375502612
ctnB(gene-MPDQ_003569)	649	537	535	2199	1970	1758	1.672726831
ctnA(gene-MPDQ_003568)	278	206	186	672	520	430	1.153910779
ctnS(pksCT)(gene-MPDQ_003567)	1364	1122	1083	4338	2813	3038	1.39922288
mrr1(gene-MPDQ_003566)	97	87	80	217	111	140	0.711356497
ctnF(gene-MPDQ_002184)	185	264	194	246	253	226	0.07
ctnR(gene-MPDQ_003570)	418	334	402	558	467	439	0.232329108
ctnH(gene-MPDQ_003573)	461	407	413	1427	1213	992	1.387317569
ctnDup1(gene-MPDQ_003575)	703	800	720	1715	1659	1306	0.96320269
