# Supplementary material for: Role of the Gene ndufs8 Located in Respiratory Complex I from Monascus purpureus in the Cell Growth and Secondary Metabolites Biosynthesis
Source: J Fungi (Basel). 2022 Jun 22;8(7):655. doi: 10.3390/jof8070655 (PMC9319538; doi:10.3390/jof8070655)
Supplement: Supplementary file 1 [file jof-08-00655-s001.zip › Table S4.pdf]

Table S4. The expression level of genes involved in fatty acid synthesis.

Symbol	WT-1_count	WT-2_count	WT-3_count	M4971-1_count	M4971-2_count	M4971-3_count	log2(fc)
FasA(gene-MPDQ_003998)	17208	18113	17171	69000	63477	59227	1.763264
FAS1(gene-MPDQ_003997)	6045	5962	5710	17818	14844	14881	1.317912
Acetyl-coenzyme-A carboxylase (gene-MPDQ_003632)	7569	6518	6994	22673	17917	16704	1.329534
FAS1(gene-MPDQ_006015)	4165	3280	3617	7987	6455	5371	0.722357
long-chain fatty acid-CoA ligase (gene-MPDQ_005101)	12698	12195	12595	20178	18387	17254	0.467942
FasA(gene-MPDQ_006016)	6245	5067	5272	10219	8967	6569	0.513919
mitochondrial 2-enoyl thioester reductase	448	500	420	655	758	641	0.48423
Short-chain dehydrogenase/reductase SDR (gene-MPDQ_003199)	67	67	79	65	100	94	0.192645
Mitochondrial beta-keto-acyl synthase (gene-MPDQ_004804)	215	184	205	139	144	130	-0.65309
FabG(gene-MPDQ_004955)	16	16	7	9	13	2	-0.85449
Long chain acyl-CoA synthetase 7 (gene-MPDQ_001984)	2320	1837	2215	1982	1846	1832	-0.27579
