# Supplementary material for: Role of the Gene ndufs8 Located in Respiratory Complex I from Monascus purpureus in the Cell Growth and Secondary Metabolites Biosynthesis
Source: J Fungi (Basel). 2022 Jun 22;8(7):655. doi: 10.3390/jof8070655 (PMC9319538; doi:10.3390/jof8070655)
Supplement: Supplementary file 1 [file jof-08-00655-s001.zip › Table S5.pdf]

Table S5. The expression level of genes involved in fatty acid degradation.

Symbol	WT-1_count	WT-2_count	WT-3_count	M4971-1_count	M4971-2_count	M4971-3_count	log2(fc)
hydroxymethylglutaryl-coenzyme A synthase （gene-MPDQ_005403）	1383	1248	1370	643	481	401	-1.51106
3-hydroxymethyl-3-methylglutaryl-Coenzyme A lyase （gene-MPDQ_004922）	123	105	100	39	54	32	-1.50674
glutaryl-CoA dehydrogenase （gene-MPDQ_000364）	1196	1053	881	560	499	415	-1.20091
long chain acyl-CoA synthetase 7 （gene-MPDQ_001984）	2320	1837	2215	1982	1846	1832	-0.27579
acyltransferase ChoActase（gene-MPDQ_001345）	3760	2917	3171	2902	2706	2271	-0.43527
carnitine acetyl transferase（gene-MPDQ_001167)	10090	9013	9283	8719	7247	6296	-0.46474
acyl-CoA dehydrogenase(gene-MPDQ_006994)	957	884	797	789	651	545	-0.52629
Acyl-CoA dehydrogenase member 10 (gene-MPDQ_001198)	217	155	188	189	200	145	-0.18535
acyl-CoA dehydrogenase(gene-MPDQ_001406)	2040	1693	1841	1713	1641	1454	-0.3224
acyl-CoA dehydrogenase family protein (gene-MPDQ_006720）	1367	1028	1008	1119	1024	788	-0.33432
enoyl-CoA hydratase domain-containing protein 2 （gene-MPDQ_000466）	707	581	613	437	322	274	-1.00025
enoyl-CoA hydratase (gene-MPDQ_001569)	6230	5165	5754	4257	3866	3513	-0.66915
3-ketoacyl-CoA thiolase (gene-MPDQ_000650)	384	269	303	163	126	98	-1.42897
acetyl-CoA C-acetyltransferase (gene-MPDQ_004118)	1861	1768	1764	1037	941	830	-1.05126
fatty-acyl coenzyme A oxidase (gene-MPDQ_005629)	383	475	489	573	530	450	0.09782
Acyl-CoA dehydrogenase(gene-MPDQ_001811）	2212	1632	1682	2808	2532	2108	0.314885
long-chain specific acyl-CoA dehydrogenase （gene-MPDQ_002811）	91	81	81	117	120	75	0.180479
