# Supplementary material for: Role of the Gene ndufs8 Located in Respiratory Complex I from Monascus purpureus in the Cell Growth and Secondary Metabolites Biosynthesis
Source: J Fungi (Basel). 2022 Jun 22;8(7):655. doi: 10.3390/jof8070655 (PMC9319538; doi:10.3390/jof8070655)
Supplement: Supplementary file 1 [file jof-08-00655-s001.zip › Table S6.pdf]

Table S6. The expression level of genes involved in PKS pathway.

Symbol	WT-1_count	WT-2_count	WT-3_count	M4971-1_count	M4971-2_count	M4971-3_count	log2(fc)
Hrpks（gene-MPDQ_006463）	95	42	68	48	46	62	-0.48543
Polyketide synthase(PfmaE)（gene-MPDQ_007933）	189	206	206	85	94	104	-1.17688
Polyketide synthase（gene-MPDQ_004128）	106	112	112	67	45	48	-1.1549
Enoylreductase（gene-MPDQ_004382）	1417	1212	1115	1432	1404	1175	-0.0118
Polyketide synthase(FUS1)（gene-MPDQ_007220）	407	331	385	324	211	234	-0.65619
Polyketide synthase(FUS1) gene-MPDQ_001325）	96	103	90	119	102	68	-0.11651
PKS1（gene-MPDQ_000613）	13	3	8	13	15	4	0.252274
Hybrid PKS-NRPS synthetase TAS1（gene-MPDQ_001039）	3839	3046	3535	8535	6071	6265	0.889487
PKS1（gene-MPDQ_006025）	6124	4489	5231	11511	8614	7716	0.694765
Hybrid NRPS/PKS enzyme（gene-MPDQ_008094）	1235	1004	1057	1559	1328	1084	0.151334
Polyketide synthase(alt5)（gene-MPDQ_006457）	144	100	114	243	207	147	0.614373
