# Supplementary material for: Role of the Gene ndufs8 Located in Respiratory Complex I from Monascus purpureus in the Cell Growth and Secondary Metabolites Biosynthesis
Source: J Fungi (Basel). 2022 Jun 22;8(7):655. doi: 10.3390/jof8070655 (PMC9319538; doi:10.3390/jof8070655)
Supplement: Supplementary file 1 [file jof-08-00655-s001.zip › Table S7.pdf]

Table S7. The expression level of genes involved in respiratory chain.

Symbol	WT-1_count	WT-2_count	WT-3_count	M4971-1_count	M4971-2_count	M4971-3_count	log2(fc)
NADH dehydrogenase, alpha subcomplex, subunit 2 (gene-MPDQ_001449)	35	37	52	32	42	28	-0.390373447
NADH dehydrogenase 1 beta subcomplex subunit 7 (gene-MPDQ_005856)	20	21	24	29	20	13	-0.200116034
NADH dehydrogenase Fe-S protein subunit 8 (gene-MPDQ_006632)	339	382	405	302	313	296	-0.405531618
NADH dehydrogenase 1 alpha subcomplex subunit 13 （gene-MPDQ_001013)	213	239	148	163	150	136	-0.525107024
NADH:ubiquinone oxidoreductase 49kD subunit (gene-MPDQ_007208)	2492	2328	2583	1658	1561	1367	-0.79957691
NADH-ubiquinone oxidoreductase 19.3 kDa subunit (gene-MPDQ_004146)	20	20	23	15	17	27	-0.166235242
NADH:ubiquinone reductase (H(+)-translocating) (gene-MPDQ_006500)	155	162	157	105	126	111	-0.570605854
NADH dehydrogenase (ubiquinone) 78K chain precursor (gene-MPDQ_004294）	6179	5089	5575	5068	4179	3730	-0.490151149
NADH:ubiquinone oxidoreductase 24 （gene-MPDQ_003677）	1570	1651	1686	1225	1287	1098	-0.548004291
NADH-ubiquinone oxidoreductase 40 kDa subunit （gene-MPDQ_000901）	5449	5224	4830	3907	3602	2781	-0.70672085
NADH:ubiquinone oxidoreductase kd subunit （gene-MPDQ_002668）	581	562	457	394	323	251	-0.844254734
NADH-ubiquinone oxidoreductase 64 kDa subunit （gene-MPDQ_007345）	2029	2680	2486	4845	5539	4342	0.929266035
NADH-ubiquinone oxidoreductase 30.4 kDa subunit （gene-MPDQ_000146）	2695	2603	2573	2144	1928	1588	-0.588778835
NADH:ubiquinone oxidoreductase 21kD subunit （gene-MPDQ_007342）	1602	1526	1471	1232	1197	963	-0.550945959
NADH dehydrogenase [ubiquinone]	4388	4096	4115	3042	2629	2181	-0.796622277

flavoprotein 1 （gene-MPDQ_001369）							
ETC complex I subunit conserved region-domain-containing protein （gene-MPDQ_003356）	1770	1610	1592	1305	1460	966	-0.530861062
Cytochrome b subunit of succinate dehydrogenase （gene-MPDQ_002964）	2865	3103	3136	2177	2467	2290	-0.491108202
Cytochrome b-c1 complex subunit 7 （gene-MPDQ_001991）	887	1010	1028	630	760	801	-0.506216068
Iso-1-cytochrome c （gene-MPDQ_004892）	5712	5702	5784	4162	3862	3301	-0.712509054
Ubiquinol-cytochrome c reductase core subunit 1 （gene-MPDQ_001959）	8993	8598	7557	6016	5593	4029	-0.803766038
Ubiquinol-cytochrome c reductase iron-sulfur subunit precursor （gene-MPDQ_007050）	3676	3500	3706	2575	2664	2173	-0.662880193
Ubiquinol-cytochrome c reductase family protein （gene-MPDQ_002598）	29	18	16	12	7	5	-1.534174391
Ubiquinol-cytochrome c reductase complex 17 kd protein （gene-MPDQ_004009）	422	404	574	350	425	501	-0.218303578
Cytochrome c oxidase copper chaperone （gene-MPDQ_005217）	48	47	37	44	44	41	-0.137045341
Cytochrome c oxidase subunit 6 （gene-MPDQ_007461）	2785	2979	3968	2915	3254	3241	-0.14331182
Cytochrome c oxidase subunit 6A （gene-MPDQ_002203）	2122	2121	2194	2444	2369	2232	0.02661361
Cytochrome c oxidase subunit 6B （gene-MPDQ_006959）	1	1	0	1	2	0	0.415037499
Cytochrome c oxidase subunit 7A （gene-MPDQ_000336）	33	18	31	13	23	22	-0.5898613
Cytochrome c oxidase subunit V （gene-MPDQ_007043）	4121	4419	4744	3653	3664	3430	-0.407597813
Cytochrome c oxidase assembly protein cox11 （gene-MPDQ_004660）	708	681	509	581	504	300	-0.583485765
Cytochrome c oxidase assembly protein cox15 （gene-MPDQ_004743）	2462	2286	2737	1623	1608	1469	-0.776193144
H(+)-transporting V1 sector ATPase	3133	2705	2709	2281	1988	1633	-0.649777544

subunit A （gene-MPDQ_007491）							
H(+)-transporting V1 sector ATPase subunit D （gene-MPDQ_000056）	267	266	268	276	240	196	-0.284245605
H(+)-transporting V0 sector ATPase subunit A （gene-MPDQ_002426）	2525	2085	2202	1735	1546	1276	-0.694910651
H(+)-transporting V0 sector ATPase subunit C （gene-MPDQ_000483）	522	468	659	437	448	439	-0.416894986
H(+)-transporting V0 sector ATPase subunit D （gene-MPDQ_005367）	552	481	487	480	427	370	-0.363599774
Mitochondrial F1F0 ATP synthase,subunit B （gene-MPDQ_002777）	4713	4252	4504	2945	2823	2357	-0.840127503
Mitochondrial F1F0 ATP synthase subunit delta （gene-MPDQ_004963）	2182	2184	1865	1675	1652	1332	-0.530317532
F1 sector of mitochondrial F1F0 ATP synthase subunit Alpha （gene-MPDQ_002787）	42414	40377	47971	33035	30778	26955	-0.635344243
F1 sector of mitochondrial F1F0 ATP synthase subunit Beta （gene-MPDQ_007042）	27868	25969	25273	19613	17547	13890	-0.747459263
F1 sector of mitochondrial F1F0 ATP synthase subunit Gamma （gene-MPDQ_000288）	6011	5903	6227	5444	4834	3923	-0.467030079
Vacuolar ATP synthase subunit B （gene-MPDQ_005728）	3871	3277	3695	4133	3878	3698	0.004975703
Vacuolar ATP synthase subunit C （gene-MPDQ_004018）	338	298	296	297	256	211	-0.402564187
V-type proton ATPase subunit E （MSTRG.7464）	497	418	360	488	373	314	-0.23770072
V-type proton ATPase 16 kDa proteolipid subunit （gene-MPDQ_001068）	23	13	21	14	21	14	-0.331075188
H(+)-transporting V-ATPase V1 sector subunit E （gene-MPDQ_004586）	298	227	242	195	218	151	-0.560312119
H(+)-transporting V1 sector ATPase subunit F （gene-MPDQ_000652）	27	18	20	26	31	32	0.35546135

V1 sector ATPase subunit H （gene-MPDQ_003364）	1020	831	788	875	633	530	-0.495016514
ATP synthase subunit D （gene-MPDQ_001488）	1277	1299	1262	1003	1017	912	-0.492875234
ATP synthase subunit E （gene-MPDQ_001047）	78	74	61	49	73	87	-0.106915204
ATP synthase subunit K （gene-MPDQ_004833）	44	33	43	28	36	32	-0.421890278
ATP synthase H chain （gene-MPDQ_006367）	558	609	472	335	333	270	-0.914584894
ATP synthase subunit G （gene-MPDQ_005570）	1623	1566	1811	1256	1338	1097	-0.545199423
Plasma membrane H+-ATPase （gene-MPDQ_007954）	1070	877	988	57	51	52	-4.303419795
ATPase complex subunit 9 （gene-MPDQ_004220）	7465	7781	8035	5698	5993	5512	-0.538056478
Mitochondrial ATP synthase epsilon chain domain-containing protein （gene-MPDQ_004432）	21	31	21	12	15	15	-0.885649888
Plasma membrane ATPase 2 （gene-MPDQ_005224）	22537	24367	26665	37930	32247	30647	0.348041709
