# Supplementary material for: Role of the Gene ndufs8 Located in Respiratory Complex I from Monascus purpureus in the Cell Growth and Secondary Metabolites Biosynthesis
Source: J Fungi (Basel). 2022 Jun 22;8(7):655. doi: 10.3390/jof8070655 (PMC9319538; doi:10.3390/jof8070655)
Supplement: Supplementary file 1 [file jof-08-00655-s001.zip › Table S8.pdf]

Table S8. The expression level of genes involved in glycolysis pathway.

Symbol	WT-1_count	WT-2_count	WT-3_count	M4971-1_count	M4971-2_count	M4971-3_count	log2(fc)
Glucokinase (gene-MPDQ_004601)	2215	2767	2972	4736	4725	4373	0.697639
Hexokinase (gene-MPDQ_007167)	4081	4975	4539	8317	8415	7232	0.713271
Glucose-6-phosphate isomerase （gene-MPDQ_007649）	11266	13951	13173	18845	19758	17221	0.437702
6-phosphofructokinase, alpha subunit （gene-MPDQ_004456）	8372	8169	7982	10160	10076	8657	0.128869
6-phosphofructo-2-kinase (6PF2K) (gene-MPDQ_005316)	3803	4155	3912	6294	6043	5429	0.476431
6PF2K-domain-containing protein （gene-MPDQ_006414）	434	411	473	490	455	487	0.020537
6PF2K-domain-containing protein （gene-MPDQ_006412）	486	520	523	461	400	298	-0.51712
Fructose-1,6-bisphosphatase （gene-MPDQ_002427）	20395	22660	22871	41965	43199	38857	0.809018
Fructose-bisphosphate aldolase （gene-MPDQ_006387）	25721	26110	24617	53374	51696	40518	0.816988
Triosephosphate isomerase （gene-MPDQ_005871）	95	97	94	88	91	76	-0.27251
Triosephosphate isomerase （gene-MPDQ_002462）	4404	3817	4046	3596	3748	3153	-0.33314
Glyceraldehyde-3-phosphate dehydrogenase （gene-MPDQ_007526）	101588	113248	103834	234455	224510	190779	0.919895
Phosphoglycerate kinase （gene-MPDQ_006673）	21620	23024	21874	35913	33396	28659	0.449659
Phosphoglycerate mutase (gene-MPDQ_002297）	14167	15306	14983	26608	27574	23530	0.700409
Pyruvate kinase （gene-MPDQ_000557）	4532	5048	4496	7309	7612	6630	0.510469
Pyruvate dehydrogenase,alpha subunit （gene-MPDQ_005291）	4109	3702	3611	4159	3815	3088	-0.16027
Pyruvate dehydrogenase E1, beta subunit（gene-MPDQ_004002）	4235	4194	4981	6605	5930	5343	0.306788
Pyruvate dehydrogenase complex dihydrolipoamide acetyltransferase component (E2) （gene-MPDQ_000229）	260	416	342	682	689	696	0.928025
Pyruvate dehydrogenase complex dihydrolipoamide acetyltransferase component (E2) （gene-MPDQ_000230）	5034	4337	4856	6202	5698	4477	0.087525
Pyruvate decarboxylase	13405	18557	13722	62972	55993	46731	1.749753

（gene-MPDQ_006259）							
Pyruvate decarboxylase （gene-MPDQ_007740）	12567	11092	12259	14136	13381	12483	0.049075
Pyruvate decarboxylase （gene-MPDQ_007222）	277	322	248	334	310	304	0.060789
Pyruvate decarboxylase （gene-MPDQ_008167）	513	382	522	494	376	389	-0.28095
Alcohol dehydrogenase I （gene-MPDQ_007350）	12014	12474	12519	27915	23855	20988	0.864516
Alcohol dehydrogenase II （gene-MPDQ_004863）	4059	3168	2825	7180	6670	4812	0.771675
Aldehyde dehydrogenase （MSTRG.8327）	26	31	43	14	18	27	-0.82922
Aldehyde dehydrogenase （gene-MPDQ_006743）	80618	62691	69665	63941	58301	47816	-0.43979
Aldehyde dehydrogenase （gene-MPDQ_001701）	17	10	16	3	5	4	-1.95031
Aldehyde dehydrogenase （gene-MPDQ_000809）	145	112	111	91	68	53	-0.92052
