# Supplementary material for: Role of the Gene ndufs8 Located in Respiratory Complex I from Monascus purpureus in the Cell Growth and Secondary Metabolites Biosynthesis
Source: J Fungi (Basel). 2022 Jun 22;8(7):655. doi: 10.3390/jof8070655 (PMC9319538; doi:10.3390/jof8070655)
Supplement: Supplementary file 1 [file jof-08-00655-s001.zip › Table S9.pdf]

Table S9. The expression level of genes involved in TCA pathway.

Symbol	WT-1_count	WT-2_count	WT-3_count	M4971-1_count	M4971-2_count	M4971-3_count	log2(fc)
Citrate synthase（gene-MPDQ_003624）	1913	1771	2028	3690	2758	2545	0.540512
Citrate synthase（gene-MPDQ_001957）	31313	31461	31255	32833	30315	24893	-0.20673
2-methylcitrate synthase（gene-MPDQ_001476）	3250	2696	3338	2527	2445	2253	-0.46823
Homoaconitase（gene-MPDQ_005976）	1690	1503	1577	1341	1068	804	-0.69216
Aconitate hydratase（gene-MPDQ_000889）	23704	25560	29604	28365	27971	25490	-0.05035
Aconitate hydratase（gene-MPDQ_006647）	1693	1449	1561	1189	1014	833	-0.74747
Aconitase iron-sulfur protein（gene-MPDQ_002397）	466	417	396	287	326	252	-0.67344
Isocitrate dehydrogenase（gene-MPDQ_007389）	5443	5151	5131	4780	4239	3641	-0.42457
Isocitrate dehydrogenase（gene-MPDQ_003844）	5665	5298	5286	5070	4728	3733	-0.37821
Homoisocitrate dehydrogenase（gene-MPDQ_000548）	1311	1154	1106	842	738	500	-0.90348
Isocitrate lyase（gene-MPDQ_005127）	14798	13940	13644	15256	14080	11890	-0.15124
Isocitrate dehydrogenase（gene-MPDQ_001022）	5393	5641	5522	5512	5403	4498	-0.21194
2-oxoglutarate dehydrogenase E1 component（gene-MPDQ_003546）	15340	16791	16088	21841	19859	17344	0.183713
2-oxoglutarate dehydrogenase E2 component（gene-MPDQ_001262）	5871	5598	5280	7127	6033	4976	-0.00053
Succinate dehydrogenase flavoprotein（gene-MPDQ_005209）	23866	23614	25507	19145	18654	16177	-0.54274
Cytochrome b subunit of succinate dehydrogenase（gene-MPDQ_002964）	2865	3103	3136	2177	2467	2290	-0.49111
Succinate dehydrogenase assembly factor 2（gene-MPDQ_005163）	295	264	251	201	232	186	-0.49496
Succinate dehydrogenase complex, subunit B（gene-MPDQ_006075）	7021	7495	9221	6801	7108	7358	-0.25372
Membrane anchor subunit of succinate dehydrogenase（gene-MPDQ_005516）	1619	1665	1896	1255	1483	1369	-0.43245
Fumarase（gene-MPDQ_004619）	10739	9921	11784	9021	8242	7210	-0.51649
Malate dehydrogenase（gene-MPDQ_006425）	17037	15468	16399	10071	9920	8466	-0.89009
Malate dehydrogenase（gene-MPDQ_006181）	1043	969	933	540	697	534	-0.83924
Malate dehydrogenase（gene-MPDQ_000227）	8302	7924	8752	7837	7553	6037	-0.33319
